# Current State of Conservation Physiology for Amphibians: Major Research Topics and Physiological Parameters

**DOI:** 10.3390/ani13203162

**Published:** 2023-10-10

**Authors:** Jun-Kyu Park, Yuno Do

**Affiliations:** Department of Biological Sciences, Kongju National University, Gongju 32588, Republic of Korea; pjk8578@smail.kongju.ac.kr

**Keywords:** amphibian, conservation strategy, research trends

## Abstract

**Simple Summary:**

Over the years, conservation physiologists have attempted to evaluate the threat factors affecting populations and manage ecosystems effectively by understanding their physiological responses. Amphibians are currently considered to be one of the most endangered vertebrate groups, and many researchers are contributing to their conservation efforts through physiological analysis. Our review aimed to examine current research trends in the conservation physiology of amphibians and identify areas for potential future studies. We categorized the 796 studies into 10 research topics. In each topic, strategies to achieve the goals of conservation physiology were identified. Additionally, we propose four comprehensive directions for future research to advance amphibian conservation physiology.

**Abstract:**

Analysis of physiological responses can be used to assess population health, identify threat factors, and understand mechanisms of stress. In addition to this, conservation physiologists have sought to establish potential management strategies for environmental change and evaluate the effectiveness of conservation efforts. From past to present, the field of conservation physiology is developing in an increasingly broader context. In this review, we aim to categorize the topics covered in conservation physiology research on amphibians and present the measured physiological parameters to provide directions for future research on conservation physiology. Physiological responses of amphibians to environmental stressors are the most studied topic, but conservation physiological studies on metamorphosis, habitat loss and fragmentation, climate change, and conservation methods are relatively lacking. A number of physiological indices have been extracted to study amphibian conservation physiology, and the indices have varying strengths of correlation with each subject. Future research directions are suggested to develop a comprehensive monitoring method for amphibians, identify interactions among various stressors, establish physiological mechanisms for environmental factors, and quantify the effects of conservation activities on amphibian physiology.

## 1. Conservation Physiology

Conservation physiology is a vital subfield of conservation biology that applies physiological concepts and techniques to comprehend and mitigate the impacts of human activities on wildlife populations and ecosystems. This field delves into physiological mechanisms associated with environmental stressors such as climate change, habitat destruction, pollution, and overdevelopment. Conservation physiologists work to establish potential management strategies to enhance resilience to environmental changes in populations by understanding the physiological responses of organisms [1,2]. This field necessitates collaboration among biologists, ecologists, physiologists, and practitioners, involving the identification of the physiological processes and mechanisms most vulnerable to environmental stressors through field experiments, laboratory research, and modeling approaches. This allows for the development of strategies to protect and restore populations and ecosystems.

Conservation physiology emerged in the early 2000s as a new field, in response to the need for integrative and interdisciplinary approaches. With roots in ecology, physiology, wildlife management, and environmental toxicology, the term was coined in 2003. The 1960s saw Rachel Carson raising awareness about human impacts on nature, while the 1990s had David Wildt researching captive animals’ reproductive biology and endocrine function. In the early 2000s, scientists began to explore physiological tools for conservation, acknowledging traditional methods’ limitations [3].

Conservation physiology has since grown rapidly, encompassing stress physiology, reproductive biology, energetics, behavior, and disease ecology [1]. Constantly evolving, it seeks practical solutions for conserving biodiversity. Conservation physiology helps identify physiological responses, assess resilience, and evaluate conservation efforts’ effectiveness. Physiological tools can be used to monitor health, stress levels, and thresholds, as well as to identify vulnerable populations. Knowledge of reproductive physiology aids endangered species breeding programs, while conservation physiology supports habitat restoration projects.

Amphibians face various threats, such as habitat destruction, pollution, diseases, and climate change, which can affect their physiology and overall health [4,5]. Conservation physiology can be used to identify amphibians’ physiological responses to these stressors, assess their resilience and resistance, and determine the effectiveness of conservation efforts in mitigating the impacts of these stressors [6]. Physiological tools can be used to monitor the health and stress levels of amphibians in degraded habitats, identify thresholds at which stress becomes maladaptive, and determine which populations or species are most vulnerable to stressors [7,8,9]. Understanding of amphibians’ reproductive physiology can also aid in the successful development of breeding programs for endangered species [10]. Conservation physiology can also be used to support the design and implementation of habitat restoration projects for amphibians. Knowledge of physiological limits for different species can help identify the most suitable populations or individuals for restoration efforts and provide insights into the timing and methods of restoration activities.

The goal of this review is to classify the topics addressed in conservation physiology research on amphibians, present an overview of the parameters measured in amphibian conservation physiology research, detect gaps in the current knowledge, and propose future research directions for amphibian conservation physiology.

## 2. Methods and Results of Data Collection

We initially retrieved a total of 4027 academic papers published between 1990 and February 2023 using “amphibian” as a search term in the academic database Web of Science. From this pool, we exclusively targeted research articles, further filtering them with the keyword “physiology”. During this filtration, we identified and excluded articles that did not directly target amphibian research. Through these meticulous steps, our dataset was refined to 796 studies specifically centered on amphibian conservation physiology (see Supplementary Material for details).

We classified 796 papers into 10 major topics and integrated them into 3 categories: (1) amphibian ecology and life history (life history and reproductive ecology, mechanisms and consequences of accelerated development, and behavioral ecology and communication); (2) environmental threats and stressors to amphibian populations (physiological responses to environmental stress, habitat loss and fragmentation, impacts of climate change, and xenobiotic exposure and effects); (3) amphibian conservation and management (disease ecology and management, conservation genetics and population ecology, and methods for studying and conserving amphibians). Two doctoral students in ecology and three doctoral researchers reviewed the 796 extracted papers and classified them into the 10 major topics. They also investigated physiological parameters in each study.

## 3. Research Topics in Amphibian Conservation Physiology

**Figure 1:**
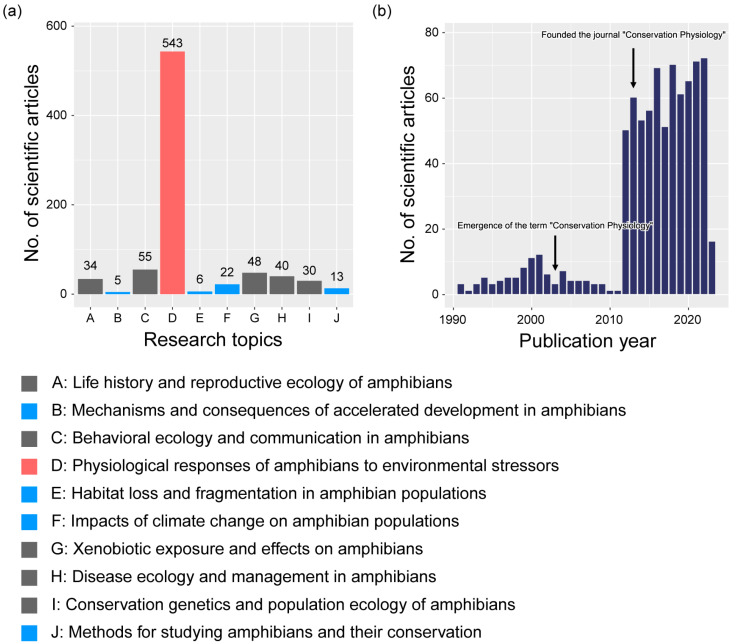
(**a**) Numbers of scientific articles delineating 10 research topics in amphibian conservation physiology. Within the bar graph, the red bars signify topics that have a significantly high count of articles. The blue bars highlight areas with fewer articles, suggesting areas that necessitate more research. The gray bars indicate topics with a medium number of research articles. (**b**) The distribution of publication years for articles centered on amphibian conservation physiology.

The primary study subjects were grouped into 10 categories after analyzing 796 research publications on the conservation physiology of amphibians. A significant majority of these papers—approximately 543, or 68.2% of the total—examined the physiological reactions of amphibians to environmental stressors. Additionally, the study articles explored various other subjects, such as behavioral ecology and communication (55 papers, 6.9%), xenobiotic exposure and effects (48 papers, 6.0%), life cycle and reproductive ecology (34 papers, 4.3%), disease ecology and amphibian management (40 papers, 5.0%), and conservation genetics and population ecology (30 papers, 3.8%). Other topics, including the consequences of rapid development, the impacts of climate change, habitat loss and fragmentation, techniques for amphibian study and conservation, and more, were also addressed (Figure 1). Given the limited research on these topics, which account for less than 3% of all research papers, we strongly recommend directing additional research efforts toward these underexplored areas. An examination of research papers related to amphibian conservation physiology published from 1991 to 2023 revealed a relatively low number of annual paper publications from 1991 to 2000, with a stable and high level maintained since 2012.

### 3.1. Amphibian Ecology and Life History

#### 3.1.1. Life History and Reproductive Ecology of Amphibians

**Figure 2:**
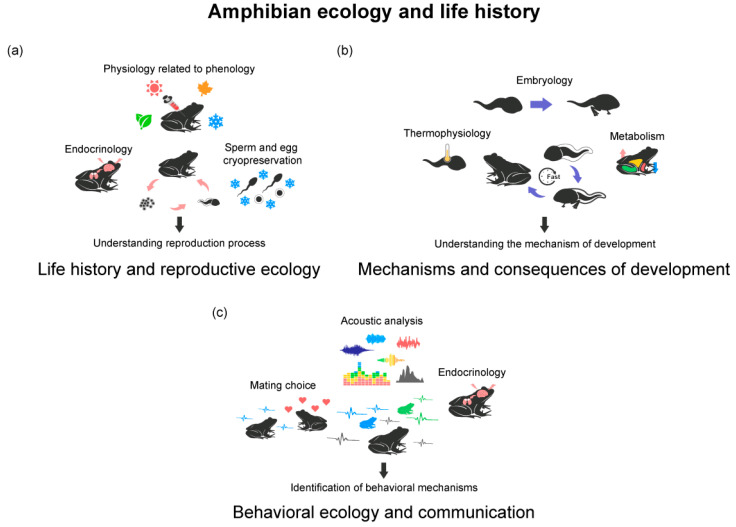
Three research topics of conservation physiology in amphibians, classified as amphibian ecology and life history: (**a**) life history and reproductive ecology; (**b**) mechanisms and consequences of accelerated development; (**c**) behavioral ecology and communication. The contents in the figure represent the analysis methods used in each research topic. The sentences under the arrows are the goals that can be achieved through conservation physiology approaches in each field.

Successful management and conservation efforts hinge on a comprehensive understanding of amphibian reproductive ecology [11]. Knowledge of breeding site characteristics, factors influencing breeding seasons, and reproductive success is vital for protecting amphibian populations [12,13]. Amphibian reproductive ecology encompasses diverse reproductive strategies, including external and internal fertilization, egg-laying, and live birth. Also, amphibians are distinguished by their complex life cycles, transitioning from aquatic larvae to terrestrial adults [14]. Researchers have studied energy and immunological investment across multiple life stages to better comprehend this intricate life history [15,16]. Also, they have studied how environmental factors such as photoperiod, temperature, and altitude influence seasonality, affecting reproductive or hibernation processes [17,18] (Figure 2a).

In recent years, hormonal therapies for inducing ovulation and in vitro fertilization have been developed in indoor breeding facilities beyond their natural habitats for conservation purposes [19,20]. The cryopreservation of amphibian sperm and eggs has also been actively pursued in conservation efforts [21,22]. Researchers have investigated endocrine compounds related to life history and reproduction, such as neuropeptide gonadotropin-releasing hormone (GnRH), luteinizing hormone (LH), and sex steroids, to understand the factors and processes influencing reproductive success and contribute to amphibian conservation [23,24].

#### 3.1.2. Mechanisms and Consequences of Accelerated Development in Amphibians

Accelerated development is a topic that many researchers are paying attention to, and efforts are underway to understand the mechanisms and impacts of this phenomenon on individuals and populations. Here, factors such as energy allocation, temperature, predation risk, and resource competition have been studied as factors that can regulate the occurrence of accelerated development in amphibians (Figure 2b).

Gilbert et al. (2020) investigated the effects of water temperature and food supply on the growth and development of the endangered species *Philoria frosti* [25]. They discovered that transformation rates rose at higher temperatures and that the availability of food also had an impact. They did, however, find that the effects of temperature and food availability on growth and development were not mutually exclusive. In the mentioned study, the authors highlighted the potential benefits of manipulating certain environmental factors, such as temperature and food supply, to enhance the rate of frog production during conservation breeding programs. This approach could reduce husbandry costs and contribute to the timely reintroduction of frogs into their natural environments.

Mueller et al. (2012) focused on the trade-off between growth and maturation and investigated the energy costs of accelerated development in amphibians [26]. Using the dynamic energy budget theory, they collected experimental data on the energy required for accelerated development in two related frog species (*Crinia georgiana* and *Pseudophryne bibronii*). They found that differences in genetic energy allocation for maturation had an impact on the difference in developmental rates between the two species. *Pseudophryne bibronii* distributed energy in the same way throughout its development, while *C. georgiana* increased the portion of energy allocated to maturation during the growth period between hatching and the start of feeding. The authors suggested that changes in energy allocation during development may be due to selective pressures to increase developmental rates, rather than being a result of developmental outcomes.

#### 3.1.3. Behavioral Ecology and Communication in Amphibians

Studies on factors shaping amphibian behavior and communication, including habitat selection, feeding, predator–prey interactions, and mate choice, have been conducted. These investigations encompass the influence of hormones and peptides on behavior, as well as pheromone use in communication. They also delve into the impacts of environmental factors like artificial noise, artificial light at night, light, temperature, and various aspects of behavioral ecology related to the senses (Figure 2c).

The male common coqui (*Eleutherodactylus coqui*) aims to attract mates. The authors of [27] found that males employ two distinct vocalizations during the mating process, with females showing a preference for one type. Similarly, Giacoma et al. (1997) discovered that male green toads (*Bufotes viridis*) emit multiple vocalizations with diverse functions, which may influence mate choice [28].

In addition to sound, visual communication is crucial in amphibian behavioral ecology. Rudh et al. (2011) studied the relationship between coloration and sexual display in strawberry poison frogs (*Oophaga pumilio*) and found that there is a significant correlation between the level of conspicuousness of a population and the sexual display behavior of males [29]. Szuroczki and Richardson (2012) found that *Lithobates sylvaticus*, which is a temporary pond breeder and does not commonly encounter fish in the wild, decreased its activity in the combined presence of a fish predator and parasites similar to when only the predator was present [30]. This suggests that, for *L. sylvaticus*, the presence of an unknown predator poses a greater threat than parasites.

The physiological regulation of these behaviors has also been studied. Carr et al. (2002) examined neuropeptides’ impact on toads’ and frogs’ feeding behavior, showing that stress-related neuropeptides can affect visual motor control and prey hunting [31]. Additionally, hormones like oxytocin, arginine, and vasopressin have been found to activate vocalizations and territorial behavior in males, influencing mate choice in females [32,33]. Researchers have also investigated physiological phenomena related to color changes during reproduction, parenting behavior, and movement [34,35].

### 3.2. Environmental Threats and Stressors to Amphibian Populations

#### 3.2.1. Physiological Responses of Amphibians to Environmental Stressors

**Figure 3:**
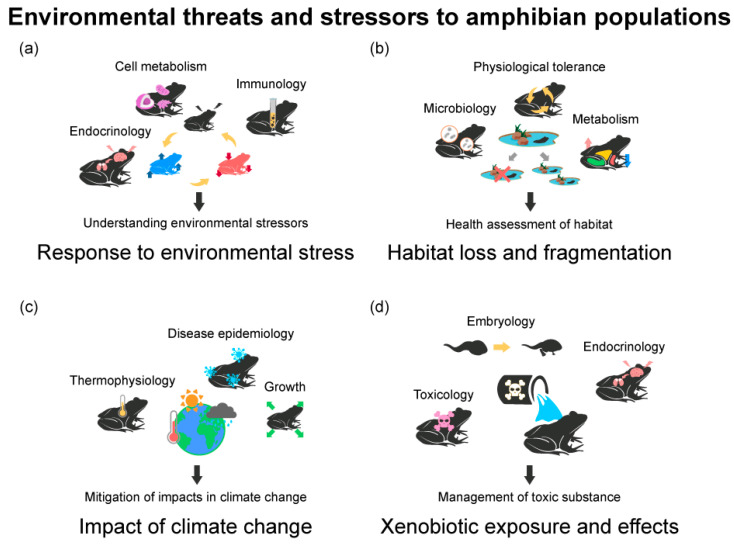
Four research topics of conservation physiology in amphibians, classified as environmental threats and stressors to amphibian populations: (**a**) physiological responses to environmental stress; (**b**) habitat loss and fragmentation; (**c**) impacts of climate change; (**d**) xenobiotic exposure and effects. The contents in the figure represent the analysis methods used in each research topic. The sentences under the arrows are the goals that can be achieved through conservation physiology approaches in each field.

The study of amphibians’ physiological reactions to environmental stressors has been a major focus of amphibian conservation physiology (Figure 3a). Various environmental elements can modify physiological stress responses, which differ between species and individuals. Amphibians attempt to adapt by exhibiting physiological responses like respiration, blood circulation, and hormone secretion [10]. However, persistent or excessive stressors can decrease their adaptive capacity, which can negatively impact species survival and reproduction [36]. In research, stress factors that are representative include temperature changes, invasive species, artificial light and noise, increasing habitat salinity, endocrine-disrupting substances, and others [7,9,37,38,39,40].

Numerous studies have monitored hormonal changes in response to environmental stress in amphibians, with glucocorticoids being the most representative hormones associated with stress [41,42]. Produced by regulation of the hypothalamic–pituitary–adrenal axis (HPA axis), these steroid hormones respond to various stressors, such as pollutants, pathogens, and changes in temperature or humidity [43,44]. Other stress-related amphibian hormones include corticotropin-releasing hormone (CRH) and arginine vasotocin (AVT), which play roles in HPA axis regulation and osmoregulation, respectively [45,46,47]. A complex interplay of hormones, neuropeptides, and signaling molecules commonly mediates the stress responses of amphibians.

Researchers are actively studying mitochondrial function, oxidative stress, inflammation, and gene expression to better comprehend environmental stress in amphibians. Mitochondria, crucial for cellular respiration and energy production, can exhibit symptoms such as metabolic shifts and reduced energy production when affected by environmental stress [48]. Environmentally induced oxidative stress in amphibian cells has prompted research on the occurrence of oxidative stress and damage to mitochondria and cell membranes [49]. Examining changes in cellular inflammation can offer insights into immune response alterations, as environmental stress can impact inflammatory responses in amphibians [50]. Investigating specific gene expression changes due to environmental stress can uncover cellular stress response mechanisms in amphibians [51]. Lately, studies have assessed individual health and physiological responses to threats using veterinary clinical examinations [8].

Environmental stress research findings are invaluable for protecting and restoring amphibian populations. Applying these results can inform suitable protective measures and strategies to reduce stress factors. Additionally, amphibian stress response research may enhance our understanding of environmental stress in other species. As amphibians serve as environmental indicators and are crucial for predicting ecosystem health, studying their responses to environmental stress contributes to ecosystem health assessment and management.

#### 3.2.2. Habitat Loss and Fragmentation in Amphibian Populations

Habitat loss and fragmentation significantly impact amphibian populations. When human activities destroy or fragment habitats, amphibian populations become isolated, affecting their evolutionary patterns and migration routes. As a result, there is a decline in amphibian species diversity, an increased risk of extinction, and a significant loss of ecosystem services [52]. Researchers study long- and short-term effects on habitats by examining factors such as endocrine hormones, symbiotic microorganisms, protein expression, and cellular metabolism (Figure 3b).

Watling et al. (2015) explored desiccation resistance as a factor influencing the distribution patterns of amphibians in tropical forests [53]. Based on their findings, they concluded that species with low desiccation resistance were less prevalent in fragmented habitats. Each species’ response to habitat fragmentation varied based on their physiological status or characteristics. Wu (2020) assessed the impact of climate change on the habitat range reduction of 91 Chinese amphibian species. The study found that 42 species were sensitive to habitat reduction, leading to increased extinction risk [54]. However, 37 species exhibited moderate sensitivity, and 12 species demonstrated high resistance. These findings emphasize the importance of species-specific research on the effects of habitat loss and fragmentation.

Goff et al. (2020) showed that habitat degradation, such as loss or fragmentation, not only affects population density and species loss but also alters amphibian microorganisms’ diversity and physiological changes [55]. Populations living in healthy habitats displayed higher survival rates, body weight, and diverse microbial communities. Conversely, polluted habitat populations exhibited poor physiological states and reduced microbial diversity. These studies indicate that amphibians’ physiological status can serve as an indicator species for assessing habitat conditions [56].

These research findings hold considerable implications for conserving amphibian habitats. Recognizing the unique effects of habitat changes on each species and developing species-specific conservation strategies are crucial. Comprehending the physiological traits of populations in healthy habitats and devising habitat restoration and management plans accordingly are also vital.

#### 3.2.3. Impacts of Climate Change on Amphibian Populations

Examining the effects of climate change on amphibian populations or communities is vital from a conservation physiology standpoint. Evaluating species- or group-specific vulnerability through amphibians’ physiological responses can guide the development of habitat restoration and conservation programs to lessen the impacts of climate change. Additionally, conservation physiology research helps identify the underlying causes of amphibian decline and offers effective conservation strategies. The related research consists of single and interaction studies on the vulnerability to climate change, physiology, behavior, and population characteristics of amphibians, as well as biological invasions, habitat loss, diseases, and other threats to amphibians (Figure 3c).

Holmes et al. (2014) suggested that precipitation and temperature play significant roles in the spread of *Batrachochytrium dendrobatidis* (*Bd*), a fungus endangering amphibians globally [57]. In dry seasons, *Bd* infection rates increase dramatically with rising precipitation, and they also grow as the average temperature during the warmest season climbs to 22 degrees Celsius. The study indicated that global warming could increase frogs’ infection risk in habitats with higher temperatures and more precipitation.

Catenazzi et al. (2014) showed that hosts under heat stress due to climate change might be highly susceptible to *Bd* infection [58]. As the elevation of the Andes Mountains rose, frogs’ thermotolerance also increased, implying that higher-elevation frogs were less sensitive to temperature increases. The study supported the alternative pathogen optimal growth hypothesis by verifying that the *Bd* infection rate grew when the frog’s growth temperature was within the optimal range.

Climate change presents a significant risk to biodiversity, particularly for species with low genetic diversity, small distribution ranges, and limited mobility. De Pous et al. (2016) demonstrated in a study of *Calotriton asper* that climate change could drastically reduce the potential distribution range of the species, affecting genetic diversity due to limited mobility [59]. This may potentially lower the physiological resilience of species to environmental factors. Moreover, numerous studies highlight that climate change’s effects on amphibians are likely to intensify the impacts of other threats, such as habitat destruction, increased pollution, and disease spread [60]. These research findings can assist in better understanding the effects of climate change and establishing suitable conservation measures. The key to amphibian conservation involves assessing species- and group-specific vulnerabilities and mitigating the impacts of climate change through proper conservation measures. This approach enables the creation of a sustainable conservation program that can address the various threats posed by climate change.

#### 3.2.4. Xenobiotic Exposure and Effects on Amphibians

Xenobiotic exposure has negative health consequences and lowers survival rates in amphibian populations (Figure 3d). Pesticides, herbicides, and heavy metals are only some of the xenobiotics that have been studied in relation to their effects on amphibian physiology [61,62].

Gabor et al. (2019) looked into the effects of glyphosate, a common herbicide ingredient, on tree frogs [63]. The study focused on exogenous corticosterone and its impact on tree frogs’ behavior, morphology, and stress hormone responses. It was discovered that glyphosate can hinder tree frogs’ adaptive predatory response, leading to population decline. Pochini et al. (2017) also found that pesticide exposure decreases amphibians’ resistance to parasitic infections by increasing parasite loads, and these effects may persist [64]. In contrast, Weeks et al. (2020) demonstrated that biocides, at realistic concentrations, do not directly harm amphibian embryos and larvae [65].

Although not an amphibian, Wagner et al. (2015) analyzed the risk posed to reptiles by pesticide exposure, particularly those in Special Areas of Conservation within the European Union [66]. They designed a species-specific risk index, considering factors such as occurrence probability, physiology, and life history traits. Their research indicated that nearly 50% of species facing higher-than-average risk from pesticide use are also under threat of global extinction. In recent years, studies have identified the effects of pollutants like heavy metals through novel metabolism analysis, biochemical tests, tissue pathology, and examination of symbiotic microorganisms [67,68]. These studies may prove beneficial for managing xenobiotic substances that pose a lethal threat to amphibians in the future.

### 3.3. Amphibian Conservation and Management

#### 3.3.1. Disease Ecology and Management in Amphibians

Amphibian diseases like chytridiomycosis (*Bd*) and *Ranavirus* pose significant threats to amphibian populations and clusters. Research into the causes, transmission, diagnosis, prevention, and treatment of these diseases aims to provide essential information and techniques for conserving amphibians and maintaining ecosystem health [69]. *Bd*-related research includes over 1000 cases, primarily focusing on physiological changes in amphibians. However, only studies using conservation physiology approaches for *Bd* and *Ranavirus* were selected.

**Figure 4:**
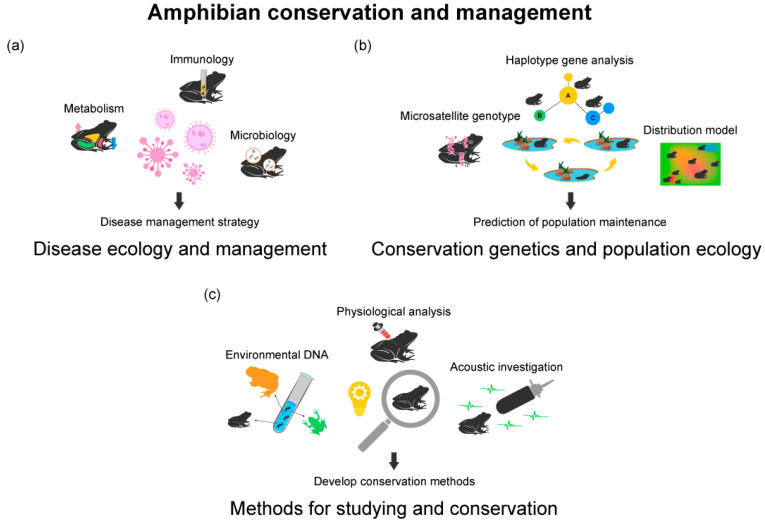
Three research topics of conservation physiology in amphibians, classified as amphibian conservation and management: (**a**) disease ecology and management; (**b**) conservation genetics and population ecology; (**c**) methods for studying and conserving amphibians. The contents in the figure represent the analysis methods used in each research topic. The sentences under the arrows are the goals that can be achieved through conservation physiology approaches in each field.

These diseases typically infect skin tissues, leading to symptoms such as skin lesions, emergence, and organ dysfunction. *Bd*, *Ranavirus*, and Iridoviridae are examples of such diseases. Red-leg disease, caused by *Aeromonas hydrophila*, is characterized by swelling or redness in the legs. Parasitic infections, particularly by helminths, play a pivotal role in amphibian health, often leading to physiological changes and developmental anomalies. For instance, chemical signals released by parasites such as leeches have been documented to cause endocrine disruption in amphibians, notably in the eastern hellbender (*Cryptobranchus alleganiensis*) [70]. Moreover, anomalies induced by trematode metacercariae infections highlight the significance of understanding the interplay between amphibian developmental stages, parasite defense mechanisms, and the resultant energy trade-offs in the host [71]. With the decline in amphibian species diversity and increased extinction threats, research on disease ecology and management strategies has become crucial [41]. Related research investigates diseases’ effects on amphibian immunity, reproduction, and heat tolerance, as well as interactions with environmental stress factors, ecological risk assessments, and the use of surrogate species (Figure 4a).

Campbell et al. (2019) examined the effects of *Bd* infection on *Litoria aurea* individuals [72]. Infected individuals exhibited high metabolic rates, low fat contents, and small testes even after the removal of the *Bd* infection. This suggests that *Bd* could have long-term effects on amphibian populations, beyond short-term lethality. Some studies have proposed rapid diagnosis or prediction methods for disease infections like *Bd*. Friday et al. (2020) provided standardized rates of disease incidence and mortality for each species in their study of salamander chytridiomycosis (*Batrachochytrium salamandrivorans*) [73]. Research like this can facilitate efficient disease management by considering species-specific traits.

Riley et al. (2013) discovered that high environmental suitability for *Bd* in southwestern Australia did not correlate with declines in frog populations [74]. Their study indicated that the impact of *Bd* might depend on innate or acquired immune status, *Bd* strain type, and *Bd* growth limitations. Consequently, the occurrence of *Bd* in some regions may not currently be a significant management concern. Although many anurans in South Korea are infected with *Bd*, which is believed to have originated there [75], most individuals or species are reported to be resistant or adapted to *Bd* and do not experience significant impacts [76].

Amphibian disease research is crucial for providing information and developing techniques to conserve populations and ecosystems. Ongoing research on disease ecology and management strategies is necessary to mitigate the threat of extinction and biodiversity loss, ultimately protecting amphibians and their ecosystems.

#### 3.3.2. Conservation Genetics and Population Ecology of Amphibians

Population decline and extinction threaten amphibians due to habitat loss, climate change, pollution, and disease [77]. Conservation genetics focuses on genetic diversity, population structure, and gene flow in amphibians [78], while population ecology studies statistical processes regulating populations, such as birth rates, death rates, and inter-population movement [79]. Research in these areas also involves genetic resource management, hormone induction in breeding systems, and suitable habitat models (Figure 4b).

Various genetic markers are used in conservation genetics research to investigate intraspecific population structure, genetic diversity, and gene flow. For instance, Murphy et al. (2000) analyzed the genetic structure of the endangered *Andrias davidianus* using microsatellite markers, finding that captive breeding programs may decrease genetic diversity [80]. In Denmark, Allentoft et al. (2009) studied the endangered *Bufo calamita* and discovered distinct and isolated populations with limited gene flow between them [81].

A recent topic in amphibian population ecology is species distribution modeling, which predicts species distribution changes based on factors like temperature and precipitation. These models can help establish conservation strategies in response to climate change [82,83]. Gerick et al. (2014) combined thermal performance experiments, species distribution models, and temperature change predictions to assess climate change risks for three amphibian species in high-altitude British Columbia [84]. The study emphasized the importance of integrating spatial and temporal changes with risk estimates.

#### 3.3.3. Methods for Studying Amphibians and Their Conservation

Amphibians play a crucial role in maintaining the health of freshwater ecosystems. Research is currently being conducted on technological advancements for amphibian survey methods, with a particular emphasis on conservation physiology approaches [85] (Figure 4c). Recently, researchers have proposed that veterinary approaches are necessary to assess the health status of amphibians, for which there is no standardized diagnostic procedure [8,86,87]. These include techniques such as X-ray imaging, blood biochemistry analysis, immunological and hormonal analyses, and histological examination. Chen et al. (2022b) proposed a technique using near-infrared spectroscopy to determine biological sex [88], while Lazcano et al. (2021) proposed the use of MRI techniques for brain research on amphibians [89]. Furthermore, studies that determine the genetic range of a population in conjunction with its physiological state are also suggested to specify management ranges [90].

Recently, new methods have been introduced to survey the distribution of amphibians. Neto et al. (2020) employed a technique that utilizes environmental DNA (eDNA) analysis to identify rare and elusive amphibians like *Cryptobranchus alleganiensis* [91]. Acoustic surveys are frequently utilized by researchers to estimate the abundance and distribution of frog populations. This method is considered to be effective and non-invasive. 

Citizen science related to amphibian surveys is actively being conducted. Amphibians’ distribution in specific regions is assessed through citizen science programs, and initiatives are undertaken to educate the public about the significance of amphibians [92,93,94]. Standardized procedures for amphibian conservation can be developed by holistically integrating these diverse methods.

## 4. Detailed Subtopics and Physiological Parameters

The 10 research topics can be classified into various detailed subtopics. These include survival and mortality rates, reproductive success, birth rates, growth rates, size, hormone levels and effects, behavior, activity patterns, nutritional ecology, diet, disease prevalence, resistance to diseases and pollutants, habitat use, distribution, physiological responses to stressors, genetic diversity, population structure, energy, metabolic rates, chemical cues, and olfactory responses, among others. We identified 12 distinct subtopics by grouping various physiological parameters under common themes (Table 1).

**Figure 5:**
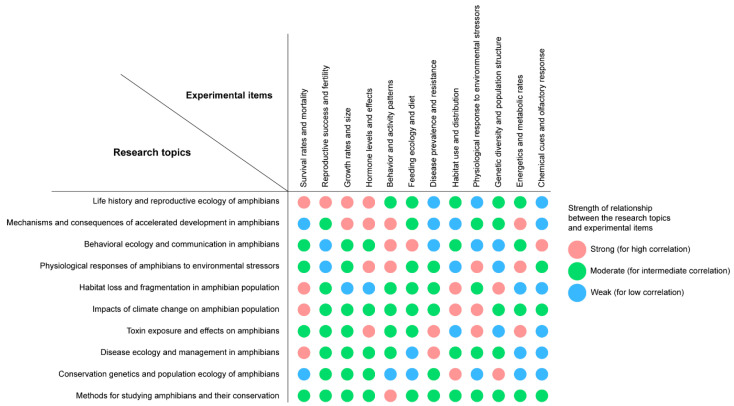
Relationships between research topics and physiological parameters in amphibian conservation physiology. High correlations are represented by red circles, intermediate correlations are represented by green circles, and low correlations are represented by blue circles.

The figure clearly highlights the qualitative strength of the relationships between the research topics and subtopics (Figure 5). These relationships are classified as strong, moderate, weak, or none. These categories are instrumental in identifying the key aspects of each research topic. Take, for instance, the research topic “Life history and reproductive ecology of amphibians”. It exhibits strong relationships with subtopics like survival rates and mortality, reproductive success and fertility, growth rates and size, and hormone levels and effects. This suggests that these subtopics form the core components of the overarching research theme. Conversely, weaker relationships exist with subtopics such as disease prevalence and resistance or chemical cues and olfactory response. This implies that these aspects may be less pivotal to the topic but still hold potential relevance.

The figure’s usefulness extends further to spotlight interdisciplinary connections among various research topics. This opens up avenues for collaboration and exploration across disciplines. For example, the topic “Physiological responses of amphibians to environmental stressors” shares strong ties with several subtopics. These are related to other research topics like energetics and metabolic rates, which also have a strong connection with “Mechanisms and consequences of accelerated development in amphibians”. It is crucial to note that the categorizations in the figure are general.

The actual strength of relationships can vary, contingent on the specific study and amphibian species. Regardless, the figure offers valuable insights into the intricate and interwoven nature of conservation physiology in amphibian research. It serves as a bedrock for future work in this field. Understanding these relationships between research topics and subtopics enables researchers to pinpoint gaps in current knowledge. It helps prioritize focus areas and contributes to a broader understanding of amphibian ecology, behavior, and conservation.

## 5. Future Directions of Amphibian Conservation Physiology

Amphibians face various threats, including habitat loss, diseases, pollution, and climate change, causing a global decline in their populations and necessitating effective conservation strategies. Conservation physiology has become an important tool for understanding amphibians’ physiological responses and assisting in conservation efforts. However, gaps in knowledge about amphibian physiology persist, and more research is needed to protect these species effectively.

To address the advancement of amphibian conservation physiology, it is essential to integrate the principles of evolutionary physiology. The success of physiological conservation relies heavily on a comprehensive understanding of physiological adaptations, tolerances, and the evolutionary trajectories of species facing conservation challenges [1]. One method to deepen this knowledge is through the comparative study of amphibians’ physiology across different taxonomic groups. A notable area of concern is the physiological characteristics of amphibians with diverse ploidy levels. The polyploid hybrid populations of water frogs, especially within the *Pelophylax esculentus* complex, are a prime example of this knowledge gap [95,96]. Additionally, understanding of the physiological traits of hybrid or mutated forms is crucial for predicting long-term evolutionary trends. Species such as *Pelophylax nigromaculatus* and *Pelophylax plancyi* often exhibit hybrid forms, resulting from mitochondrial genome import events [97,98]. However, the physiological traits of these species with altered mitochondrial DNA are not well understood. Given that hybrid forms are frequently linked to decreased survival rates and morphological anomalies [99,100], it is vital to determine the potential physiological consequences of these forms. Finally, there is an urgent need to expand our understanding of physiological tolerance in relation to conservation threats. For example, amphibian populations are significantly impacted by the salinity resulting from road deicing [101,102,103]. Investigating their adaptive strategies or resilience to such salinity changes might provide valuable insights for conservation efforts [104,105].

**Figure 6:**
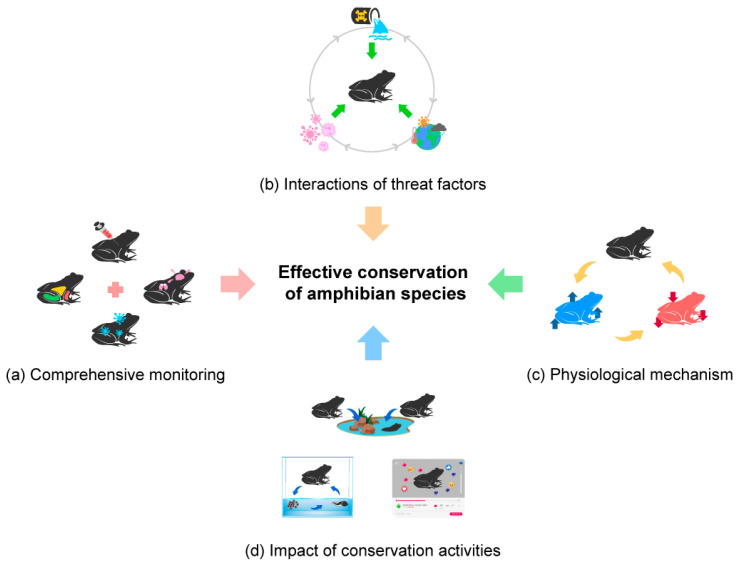
Four research topics that need to be investigated to effectively conserve amphibians through a conservation physiology approach: (**a**) A comprehensive amphibian monitoring program, including physiological measurements, should be developed. (**b**) In addition to responses to single stressors, we need to understand the interactive effects of multiple stressors. (**c**) The physiological mechanisms of amphibians, as well as their simple physiological responses to environmental factors, must be understood. (**d**) Quantification of physiological conditions in amphibians based on the impact of conservation activities and measures is needed.

Another crucial research area is developing comprehensive monitoring programs for amphibians. These programs provide valuable information on population status and help confirm declines before they become irreversible. However, current programs often focus only on population numbers, neglecting physiological measurements. By incorporating physiological measurements like stress hormone levels or immune function, we can better understand amphibians’ physiological responses and aid in conservation efforts (Figure 6a).

Another important research area is investigating the interactions between multiple stressors on amphibians. Amphibians often face several stressors simultaneously, such as habitat loss, pollution, and diseases, but the interaction effects on their physiology are not well understood. For instance, pollution may weaken immune function, making amphibians more susceptible to diseases. Climate change can alter the timing of breeding and migration, negatively impacting populations’ survival. Understanding these interaction effects can lead to better predictions and more effective conservation strategies (Figure 6b).

Additionally, more research is needed on the physiological mechanisms of amphibians’ responses to threats. Some amphibians can withstand high pollutant levels, while others are highly sensitive. Understanding these differences can help identify crucial survival characteristics in polluted environments. Similarly, investigating physiological mechanisms related to disease resistance can help in developing protective strategies against new diseases (Figure 6c).

Lastly, the effects of conservation activities on amphibian physiology require further study. Conservation actions such as habitat restoration or artificial breeding programs can have physiological impacts on amphibians, like changes in immune function or stress hormone levels. Understanding of these effects is essential for evaluating interventions’ effectiveness and minimizing negative impacts on amphibians (Figure 6d).

## Figures and Tables

**Table 1:** Physiological parameters extracted from conservation physiology research on amphibians. Various physiological parameters were extracted from 12 detailed subtopics.

Category	Physiological Parameters (Experimental Items)
Survival rates and mortality	Annual survival rate; cause-specific mortality rate; age-specific mortality rate; mortality due to predation, disease, or environmental stressors; survival and mortality of different life stages (e.g., eggs, larvae, juveniles, adults)
Reproductive success and fertility	Clutch size; hatching success; fertilization success; brood size; nesting success; incubation period; mating success; reproductive lifespan
Growth rates and size	Growth rate (length or mass); age-specific growth rate; size at metamorphosis; size at maturity; growth plasticity in response to environmental conditions
Hormone levels and effects	Hormone levels (e.g., testosterone, estrogen, corticosterone); hormone receptor density; behavioral effects of hormones (e.g., territoriality, courtship, aggression)
Behavior and activity patterns	Activity budget (e.g., proportion of time spent foraging, resting, moving); circadian rhythm; social behavior (e.g., mating, parental care, territoriality); response to stimuli (e.g., predator avoidance, conspecific recognition)
Feeding ecology and diet	Diet composition; feeding rate; prey selection; digestive efficiency; nutrient absorption and allocation
Disease prevalence and resistance	Disease prevalence (e.g., prevalence of chytrid fungus in amphibians); resistance to diseases; immune response (e.g., cytokine levels, leukocyte counts)
Habitat use and distribution	Habitat selection; home range size; migration patterns; habitat fragmentation effects on population dynamics
Physiological responses to environmental stressors	Thermal tolerance; response to temperature fluctuations; resistance to pollutants; detoxification enzyme activity
Genetic diversity and population structure	Genetic diversity (e.g., heterozygosity, allelic richness); effective population size; gene flow; population structure (e.g., subpopulations, metapopulations)
Energetics and metabolic rates	Basal metabolic rate; maximum metabolic rate; energy intake and expenditure; respiration rate; energy allocation to growth, reproduction, and maintenance
Chemical cues and olfactory responses	Detection threshold for chemical cues; discrimination ability between different chemical cues; behavioral response to chemical cues (e.g., foraging, predator avoidance, mate choice)

## Data Availability

Not applicable.

## Supplementary Materials

The following supporting information can be downloaded at: https://www.mdpi.com/article/10.3390/ani13203162/s1. Manuscript-supplementary.csv.
